# Exploring the Spatial Association between Social Deprivation and Cardiovascular Disease Mortality at the Neighborhood Level

**DOI:** 10.1371/journal.pone.0146085

**Published:** 2016-01-05

**Authors:** Mary Margaret Ford, Linda D. Highfield

**Affiliations:** 1 St. Luke’s Episcopal Health Charities, Houston, Texas, United States of America; 2 Department of Management, Policy & Community Health, University of Texas School of Public Health, Houston, Texas, United States of America; Universität Bochum, GERMANY

## Abstract

Cardiovascular disease (CVD), the leading cause of death in the United States, is impacted by neighborhood-level factors including social deprivation. To measure the association between social deprivation and CVD mortality in Harris County, Texas, global (Ordinary Least Squares (OLS) and local (Geographically Weighted Regression (GWR)) models were built. The models explored the spatial variation in the relationship at a census-tract level while controlling for age, income by race, and education. A significant and spatially varying association (p < .01) was found between social deprivation and CVD mortality, when controlling for all other factors in the model. The GWR model provided a better model fit over the analogous OLS model (R^2^ = .65 vs. .57), reinforcing the importance of geography and neighborhood of residence in the relationship between social deprivation and CVD mortality. Findings from the GWR model can be used to identify neighborhoods at greatest risk for poor health outcomes and to inform the placement of community-based interventions.

## Introduction

Cardiovascular Disease (CVD), the leading cause of death in the United States (US), has contributed annually to over half a million deaths in the country for the past two decades [1,2]. It is estimated that nearly half of all Americans are at risk for CVD due to modifiable risk factors including high blood pressure, high cholesterol, and smoking [2]. Disparities in CVD mortality have remained persistent in the US over the past decades despite efforts to slow the trend in mortality [3,4]. Regional variations within the US show the highest rates of CVD morbidity and mortality are experienced in the “stroke belt,” which consists of the Southeastern coastal states from Texas to Virginia [2,5,6]. These observed geographic disparities are likely perpetuated by the economic and educational disadvantage that is simultaneously experienced in these states [7].

Considerable research has supported the biological pathway linking socioeconomic disadvantage to the physical manifestations of CVD. Stress, depression, and social isolation have all been linked to CVD, and in particular anxiety is associated with heart disease on levels comparable to smoking and hypertension [8]. Poverty and neighborhood of residence, two geographically-linked factors, have been tied to CVD mortality and its risk factors, with persons living in communities that aren’t conducive to healthy lifestyle choices being at higher risk of CVD [9–11]. The urban poor may be exposed to elevated environmental stress as a result of overcrowding, street violence, or the residential segregation that experienced in many cities [12]. High concentrations of impoverished or minority populations within neighborhoods often perpetuate health disparities as residents of segregated neighborhoods are less likely seek health services [13,14].

In contemporary epidemiological research, social deprivation refers the effect or consequence of lack of income and resources such as healthful diet, adequate living conditions, and participation in social activities[15]. (e.g. social norms) In order to quantify social deprivation, deprivation indices have been developed and validated by researchers to create universal measures that go beyond simply using income as a proxy for disadvantage. Deprivation indices are typically used to identify geographic areas of highest social and economic need. While widely used in countries such as the United Kingdom (UK)[16–18], within the US deprivation is not a measure commonly used by researchers or policymakers in public health decision-making. In order to best inform future interventions it is important to understand the neighborhood-level distributions of and associations between these factors.

Given that place plays a key role in the distribution of both CVD mortality and its known risk factors, geographic variability must be accounted for when modeling the relationship between CVD and social deprivation. In global regression models, such as Ordinary Lease Squares (OLS), a global parameter estimate is applied to an entire dataset, which does not account for regional spatial variation in the association. Relationships that are non-stationary can result in problems with parameter estimates or interpretation of the model [19,20]. Geographically Weighted Regression (GWR) was developed as a method to extend traditional regression techniques by accounting for the presence of spatial non-stationarity in relationships between the variables [19].

In the present study, the authors examine the spatial association between CVD mortality and social deprivation in order to further understanding of neighborhood-specific associations by accounting for spatial non-stationarity. The geographically-weighted modeling approach will enhance a more traditional regression models’ estimates to provide a more complete description of the underlying relationship. Moreover, the results identify specific neighborhoods that may gain the greatest benefit from place-based public health interventions that address modifiable CVD risk factors. While methods exist to model social deprivation and model spatial relationships between a number of diseases, including cardiovascular disease [21], to-date the authors are not aware of research published within the US that has explored the spatial relationship between CVD mortality and social deprivation. The application of the methods and results of this study will add a powerful approach for health researchers to complement traditional regression techniques and adopt more meaningful examinations of geographically influenced health outcomes and predictive factors.

## Methods

### Site Selection

**Fig 1:**
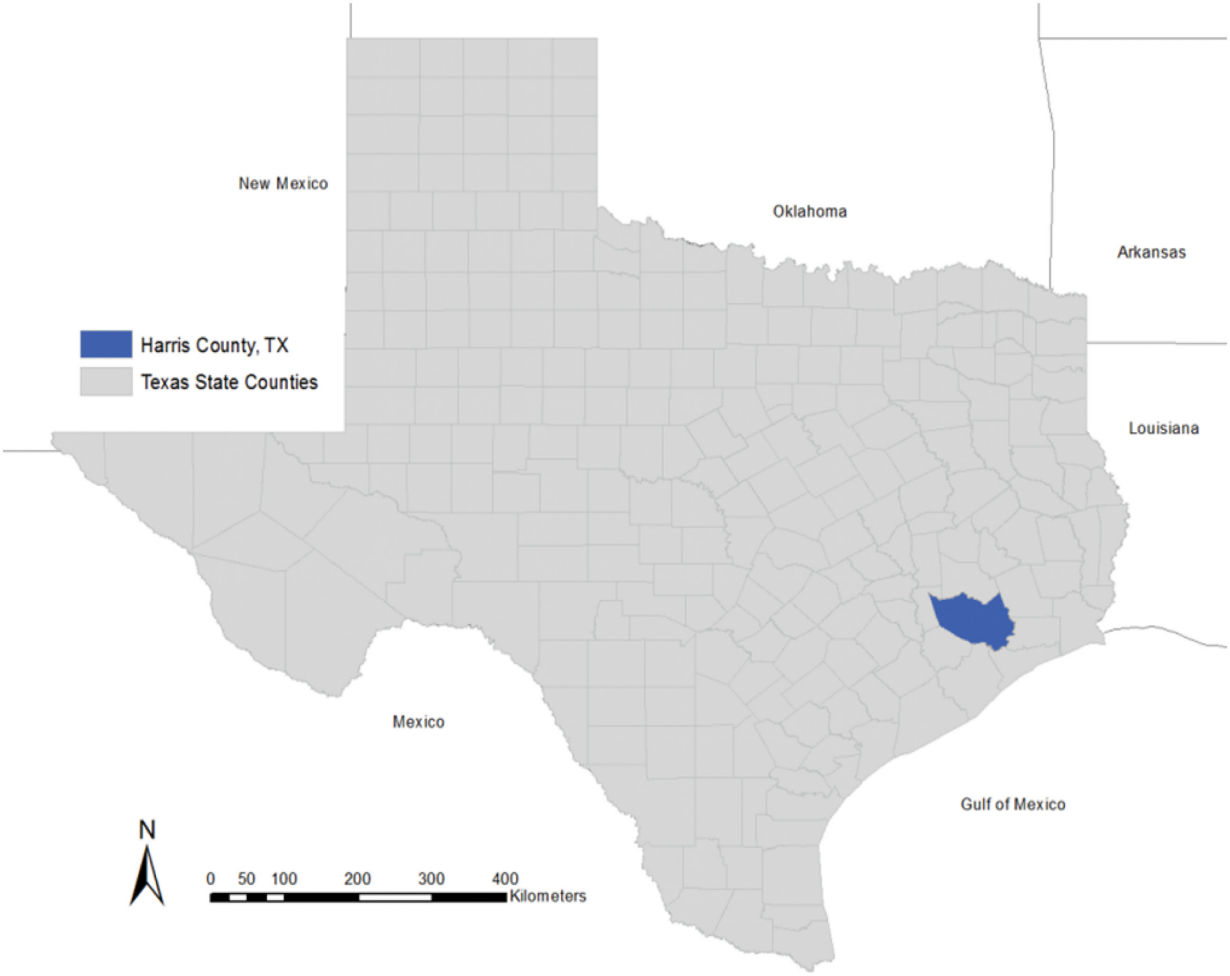
Harris County, Texas

Harris County, Texas, which contains Houston, Texas, the fourth largest city in the US, was selected as the study site due to its diversity in income, race, and population density. [Fig 1] With over 4.2 million inhabitants, much of Harris County is considered a densely urban Metropolitan Statistical Area (MSA). Houston, TX is a majority-minority city with 41% of the residents identifying as Hispanic or Latino and 19.5% identifying as black or African American in 2012 [22]. Census tracts [23] were selected as the geographic unit in this study as this unit has been shown to be suitable for use in analyses with area-based measures of deprivation [24]. Census tracts reflect the smallest geographic unit of analysis possible to simultaneously maintain resolute spatial variation and an adequate population size for stability in the rate calculations.

### Cardiovascular Disease Mortality Rates and Spatial Autocorrelation

CVD mortality crude rates were calculated by census tract using US Vital Statistics records’ mortality data [6] for the years 2008–2010. A three-year aggregate crude rate [25,26] was used to get a reliable rate for each census tract by having a minimum of 15 deaths per tract. Prior to use in the model, the rates were evaluated for normality by generating histograms. Spatial autocorrelation in the geographic distribution of CVD mortality rates were evaluated using a global Moran’s I test. The Moran’s I test measures the degree to which disease pattern is clustered, dispersed, or randomly distributed across census tracts by computing the deviation from the mean for each tract [27]. A Moran’s I value greater than zero indicates positive spatial autocorrelation or clustering, and a value less than zero indicates negative autocorrelation or dispersion.

### Townsend Deprivation Index

The Townsend Index was selected as the deprivation index to measure social deprivation in this study. The Townsend Index, a widely-used measure of social deprivation and disadvantage, was created by Peter Townsend, et al in 1988 in the UK to compare levels of deprivation across Census Output areas or postcodes [15,28]. The index may be applied at any level of geography; used here to examine US census tracts. [29]. The index is comprised of four variables: percent of residents unemployment, percent of houses with non-car ownership, percent of households not owner occupied, and percent of households with over-crowding. US census data were used to calculate these variables from Geolytics’ 2009 dataset [30]. The four variables were transformed with a natural log to eliminate skewness [31]. Composite scores were generated by calculating a z-score for the four variables, with the four z-scores being summed to create a Townsend score. The final Townsend scores were broken into quintiles to rank each census tract for the Index, with 1 representing the lowest local social deprivation and 5 representing the highest level of local social deprivation.

### Covariate Selection

Covariate selection was based on a review of the literature for sociodemographic risk factors associated with CVD mortality and available data. The covariates were included to control for population-level confounding between social deprivation and CVD mortality and other risk factors within census tracts. Variables were evaluated for normality and were log-transformed if needed based on the results of the linearity tests. Covariates established through the literature review for inclusion in the model were age, median household income (MHHI) by race, and education level[3,32–35]. Age was broken into five continuous variables of the proportion by census tract for the age groups (0–19, 20–34, 35–49, 50–64, and 65+). Income by race variables were the proportion of the census tract population by MHHI for the races of white non-Hispanic, Hispanic, black non-Hispanic, and Asian. The MHHI by race variables were divided by 1,000 to produce results by $1,000 increases in income. The four education variables were built as proportions by census tract for No High School Degree, High School or Some College, Bachelors or Associates Degree, and Graduate Degree.

### Ordinary Least Squares and Geographically Weighted Regression Model Building

An Ordinary Lease Squares (OLS) Regression model was fit to the data to test for statistical significance and association at a global level. The OLS model was built as a first-step in the model building process to establish global significance (p < .05) between CVD and the predictor variables across the study site. The results of the OLS model informed which variables were included in the local, GWR model [36,37]. A backwards, stepwise regression model was built manually and validated against an automated stepwise function to produce the model with best-fit. OLS models with statistically significant variables were compared using adjusted R^2^ values and F-tests. Covariates were assessed with a Variance Inflation Score (VIF) after each model run and were dropped based on least significance (greatest p-value) and if found to produce high collinearity (VIF>10) within the model.

Following the identification of the best-fit OLS model, a GWR model was built including the variables identified in the OLS model. GWR is a method of regression modeling that that quantifies spatial nonstationarity on a local level that cannot be captured in a global model [19,20]. GWR captures this spatial variability by calibrating a multiple regression model, testing different relationships at different geographic points. The use of GWR increases the capacities of regression techniques by taking into account spatial structure and allowing local examination of parameters [20,38]. A Gaussian linear model with an adaptive bi-square geographic kernel was used. The “golden section search” function for optimal bandwidth selection was used, using Akaike Information Criterion (AIC) as the criterion [39]. The residuals from the GWR model were tested for spatial autocorrelation using the global Moran’s I statistic to determine whether the assumptions of regression were being met [40]. To assess the model fit between the analogous OLS and GWR models, the global R^2^ of the OLS model was compared to the GWR local R^2^, with a higher R^2^ indicating greater explanation of variance in the model. In addition to the R^2^, the AIC was used to compare the two model types [20,37,38]. To further interpret the GWR model the regression coefficients for each variable were mapped to assess spatial variation in the parameters and to locate neighborhoods where the significant explanatory variables exhibit little, strong, or no regional variation. The local R^2^ values were mapped to identify locations where either the model explains a high proportion variance or where it does not and further investigation may be warranted.

All statistical tests were conducted in Stata 11 (Stata Corp., College Station, TX [41]). Choropleth maps and Moran’s I statistics were generated using ArcGIS 10. GWR analyses were conducted in GeoDa’s GWR4 software [42],

## Results

**Fig 2:**
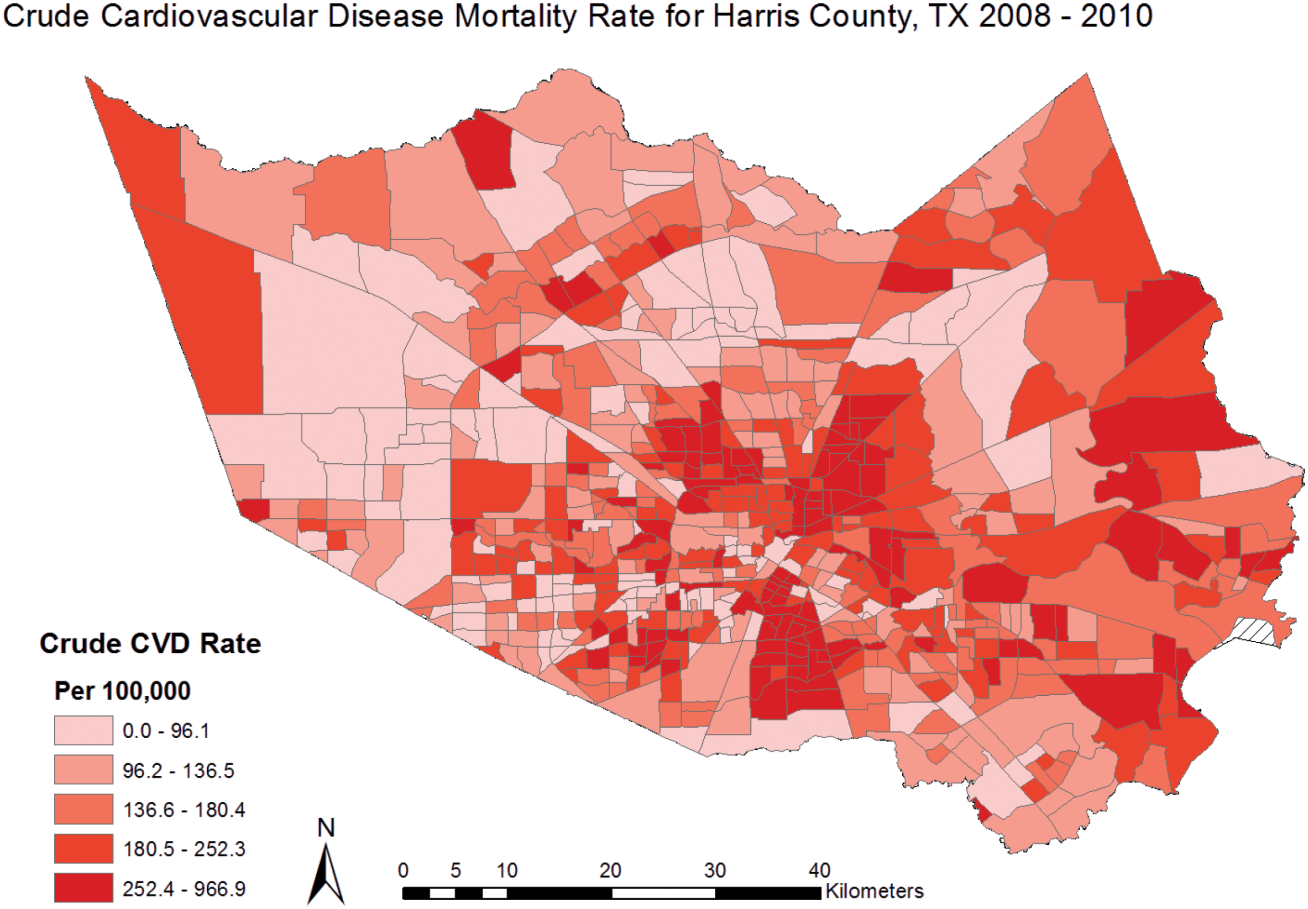
Crude CVD Mortality Rates for Harris County, TX 2008–2010.

**Fig 3:**
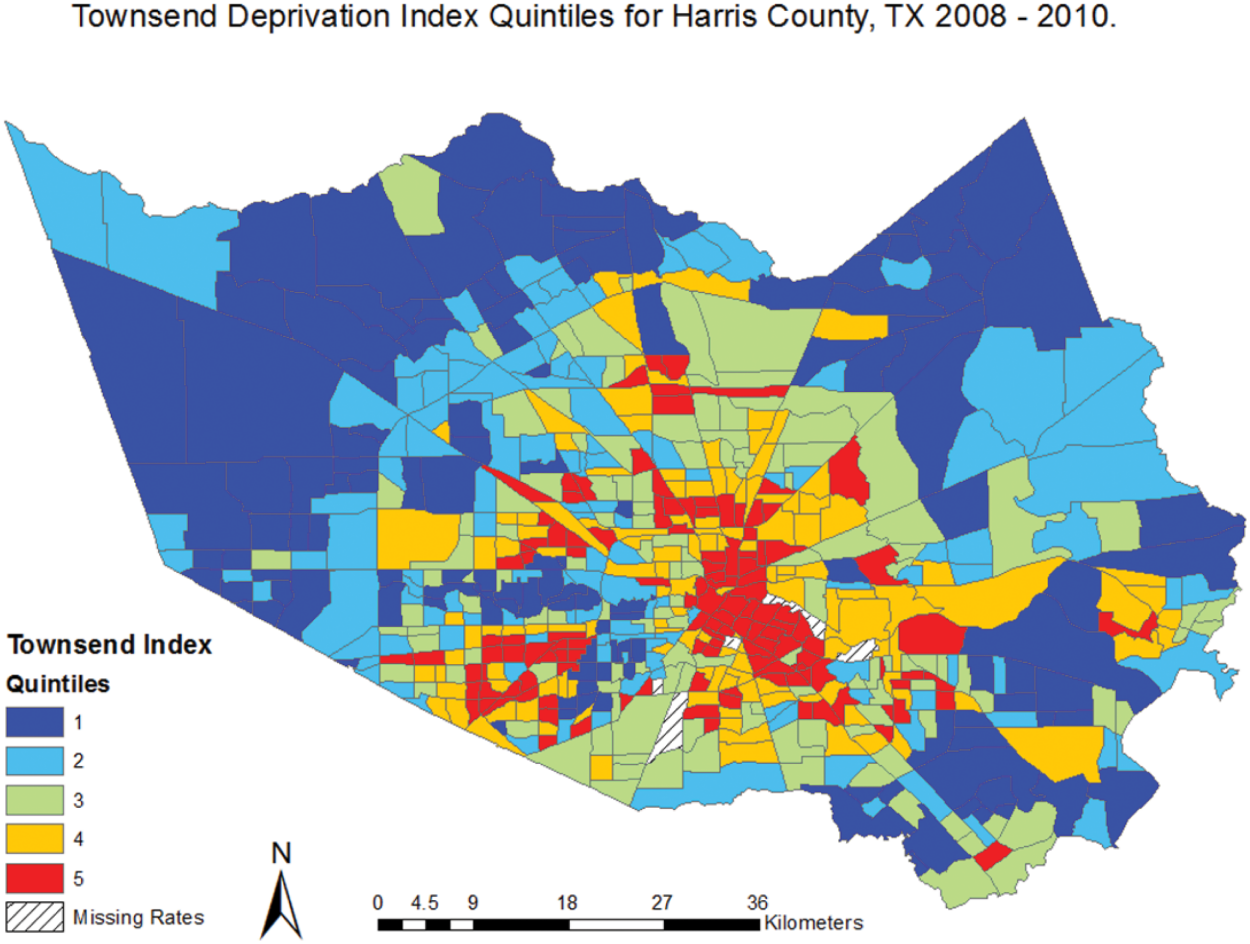
Townsend Deprivation Index Quintiles for Harris County, TX 2008–2010.

Cardiovascular disease mortality rates were calculated for 649 census tracts in Harris County with a minimum rate of 15.5 deaths per 100,000 and a maximum rate of 967 deaths per 100,000. [Table 1] Clustering of high CVD mortality rates can be visually identified in the central region of Harris County, representing the inner-city area of Houston. [Fig 2] The global Moran’s I for CVD mortality was .11 (z = 20.59, p<0.01), indicating low but significant spatial autocorrelation. Given the z-score for the Moran’s I test of 20.59, there was a less than 1% likelihood that the spatial clustering observed in the CVD mortality rates is the result of random chance. A Townsend Index score was produced for 644 census tracts in Harris County, ranging from 1–5. An Index score was not calculated for five tracts due to null values in the census tract covariate data used to build the index (one or more of the necessary index variables was missing for these census tracts). The distribution of high and low index scores is shown in Fig 3. Descriptive statistics were produced for all covariates and are reported on for those that were found to be statistically associated with CVD mortality. [Table 1]

**Table 1:** Descriptive Statistics for Outcome, Exposure, and Control Variables.

	Minimum	Maximum	Mean	Std. Deviation
CVD Mortality Rate	15.5	966.9	183.9	115.0
Townsend Index	1.0	5.0	3.0	1.4
Age 65+	0.0	0.3	0.1	0.0
Income, Black ($1000)	2.2	201.8	39.1	25.6
Income, Asian($1000)	2.2	201.5	43.4	37.7
Education	0.1	1.0	0.5	0.1

The OLS regression model found a statistically significant association (p < .01) between CVD mortality and social deprivation. [Table 2] For every increase in quintile within the Townsend Index, an increase of 25.36 CVD deaths is expected when holding age, income by race, and education constant. The covariates found to be statistically significant in the model were ages 65 and over, income for black race, income for Asian race, and the education variable of High School degree and Some College. [Table 2] The R^2^ for the final OLS model was .57, indicating 57% of the variance seen in CVD mortality is explained by variables in the model. The R^2^ value signifies a strong model fit and explanation of variance. The F-test for the best-fit OLS model was 171.71, p<0.02, and the Akaike’s Information Criterion (AIC) score was 16736.76.

**Table 2:** Ordinary Least Squares Regression Model for CVD Mortality and Social Deprivation, holding age, income by race, and education constant.

	Beta	Std. Error	95% Confidence Interval	p-value
Townsend Index	25.4	2.8	(19.90, 30.83)	0.00
Age 65+	1900.8	69.6	(1764.05, 2037.45)	0.00
Income, Black	0.3	0.1	(0.04, 0.59)	0.02
Income, Asian	-0.4	0.1	(-0.56, -0.20)	0.00
Education	184.8	24.9	(135.90, 233.67)	0.00

**Fig 4:**
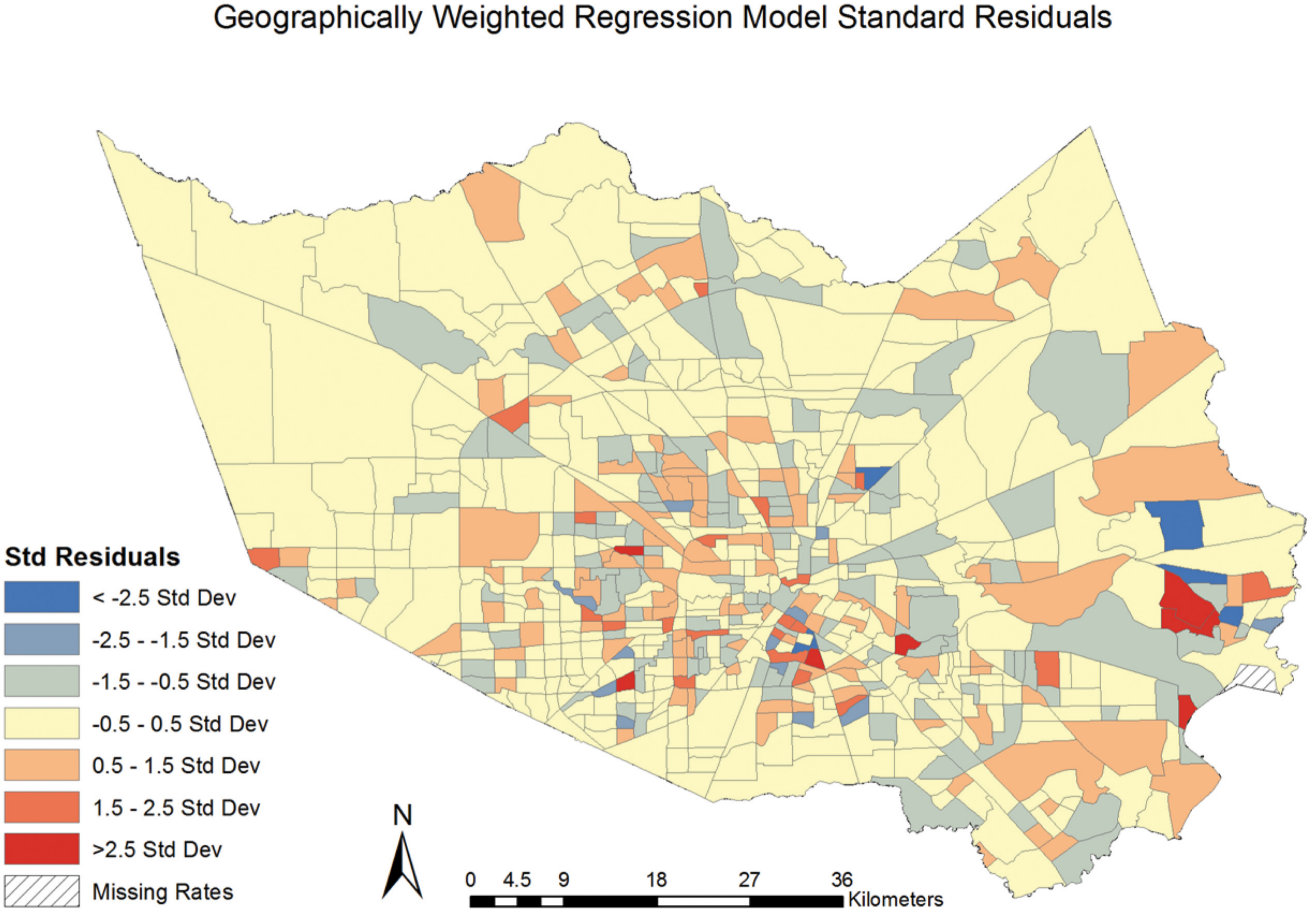
Geographically Weighted Regression Model Studentised Residuals.

The GWR model was built with the same covariates as in the best-fit OLS regression model[36,37,43]. The results of the GWR model found significant (p < .01) spatial heterogeneity between CVD mortality and social deprivation The model’s R^2^ value of 0.64 indicates 64% of the variance seen in CVD mortality is attributable to variables in the model. The studentised residuals were mapped by census tract, identifying census tracts that were spatially heterogeneous (ie. non-stationary). [Fig 4] The map of the studentised residuals identifies areas, shown in blue or red, where the model under or over (respectively) predicted the values of CVD mortality. Values between -.5 and .5 indicate census tracts where the GWR model best predicted values. The pattern of blue and red in the studentised residuals appears to be random, suggesting the GWR model has accounted for the spatial autocorrelation in the association. The AIC for the GWR model was 7382.9.

**Fig 5:**
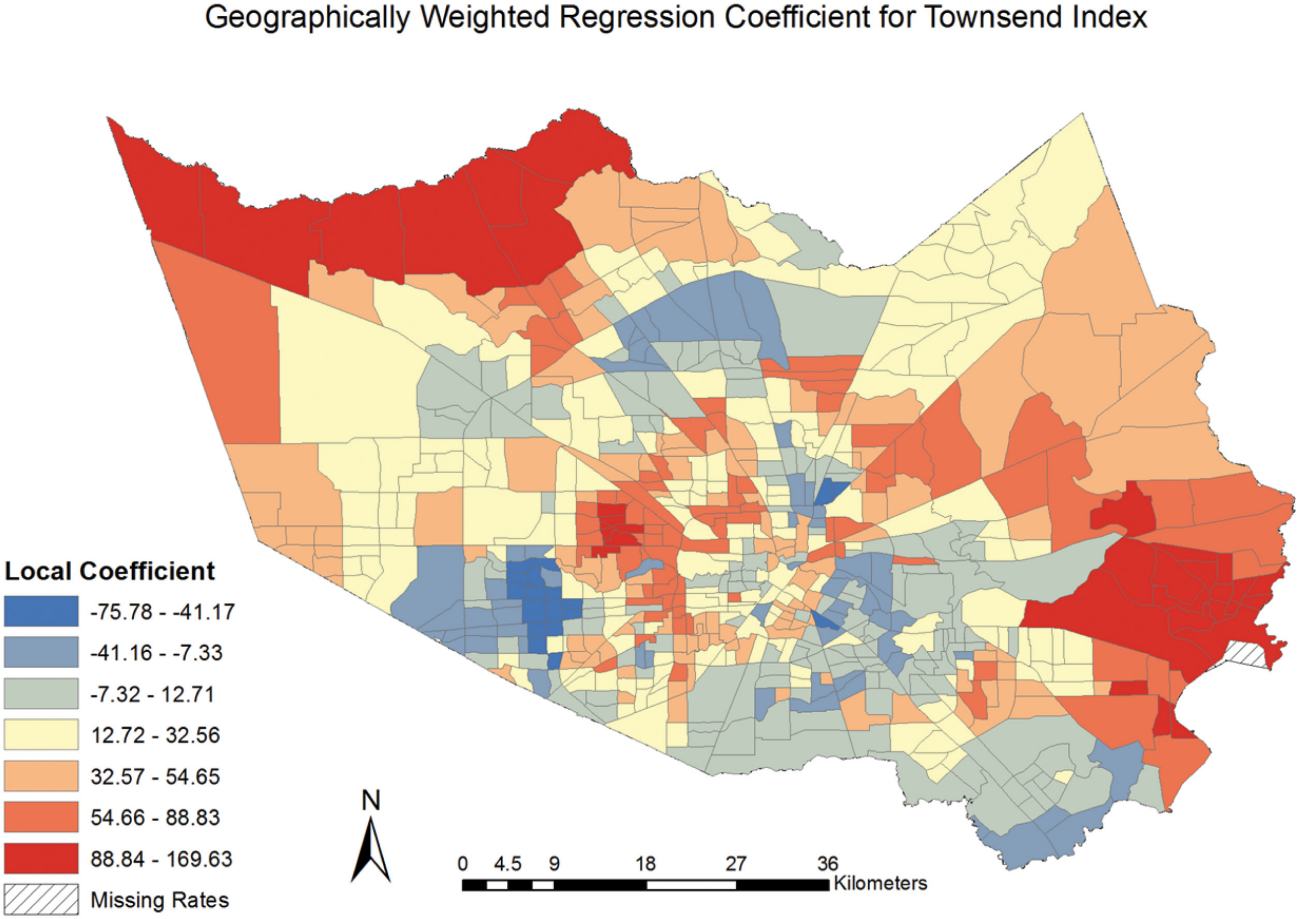
Geographically Weighted Regression Coefficient for Townsend Index.

The local regression coefficients representing the strength of association between CVD mortality and social deprivation were mapped, [Fig 5] revealing the spatial variation in the relationship. The coefficients identify higher values in the central, more urban area of Harris County, TX comprising the City of Houston. We find that the strength of the association varied across the county, with a negative association being experienced in some neighborhoods. This finding highlights the capabilities and benefits of using GWR to reveal differing and, in some cases, unexpected variation in the relationship between CVD mortality and social deprivation resulting from spatial heterogeneity.

**Fig 6:**
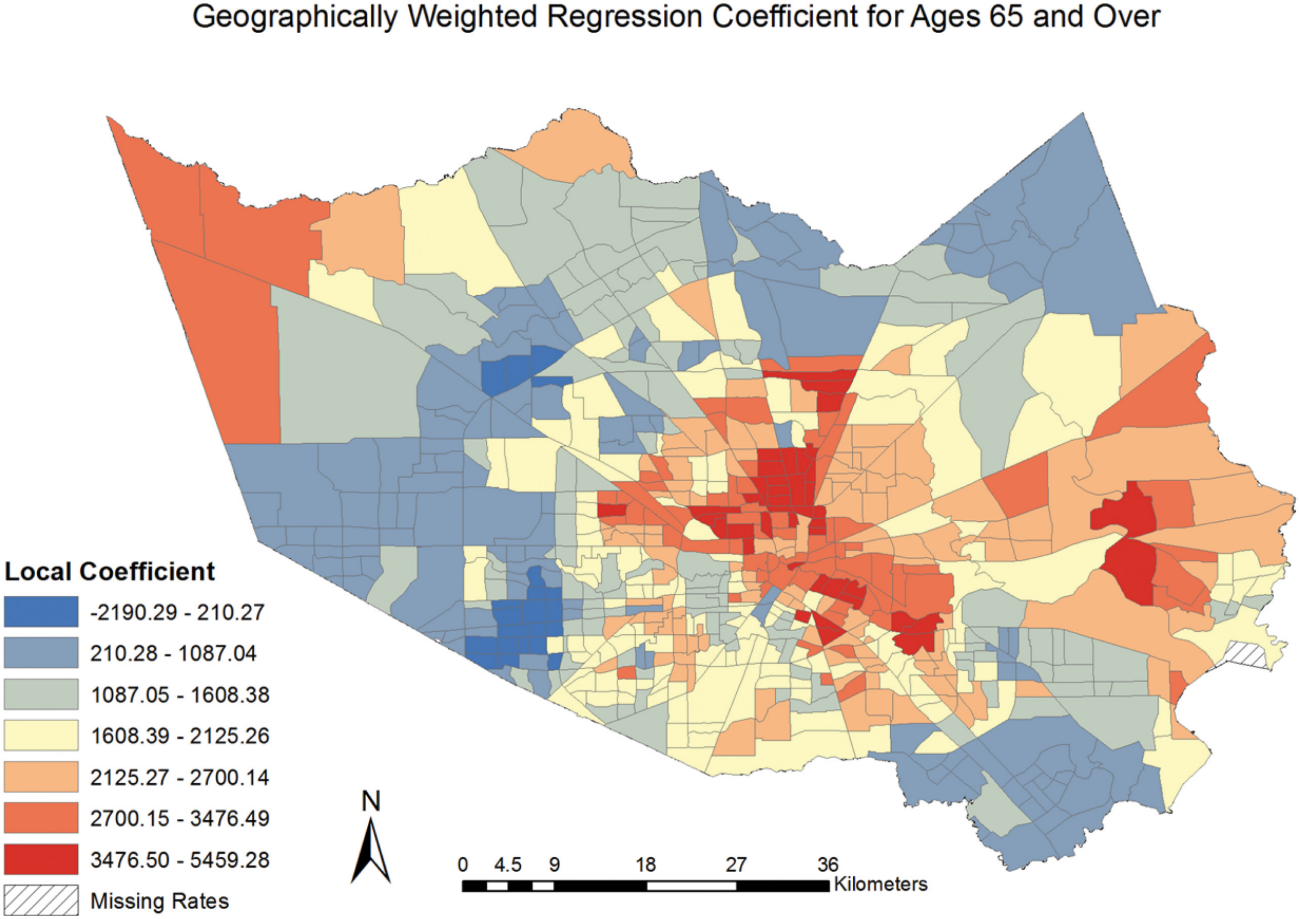
Geographically Weighted Regression Coefficient for Ages 65 and Over.

**Fig 7:**
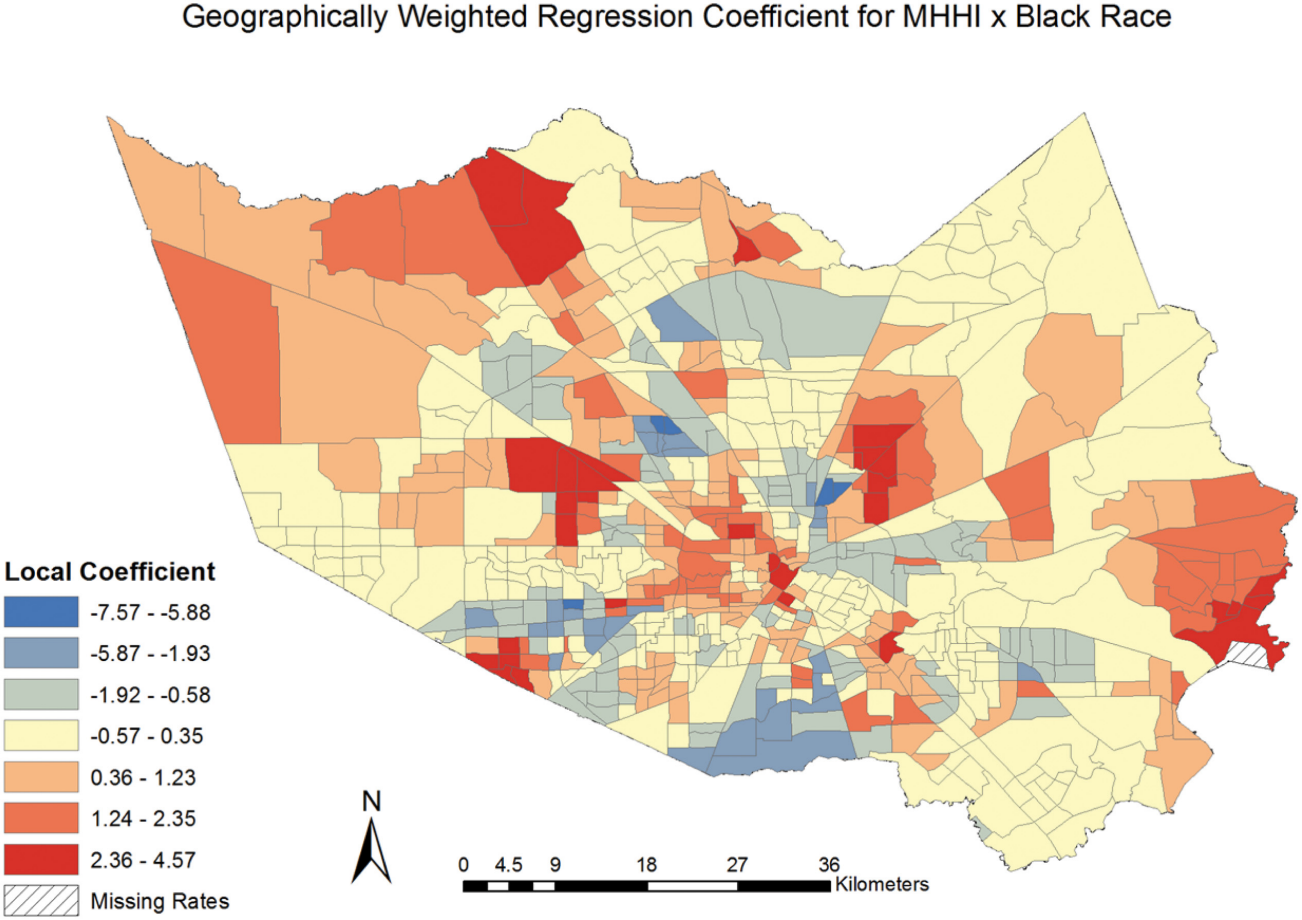
Geographically Weighted Regression Coefficient for Income by Black Race.

**Fig 8:**
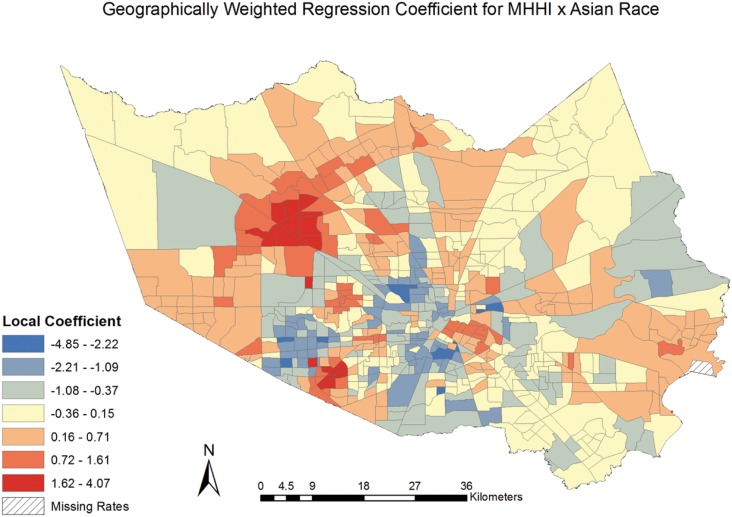
Geographically Weighted Regression Coefficient for Income by Asian Race.

**Fig 9:**
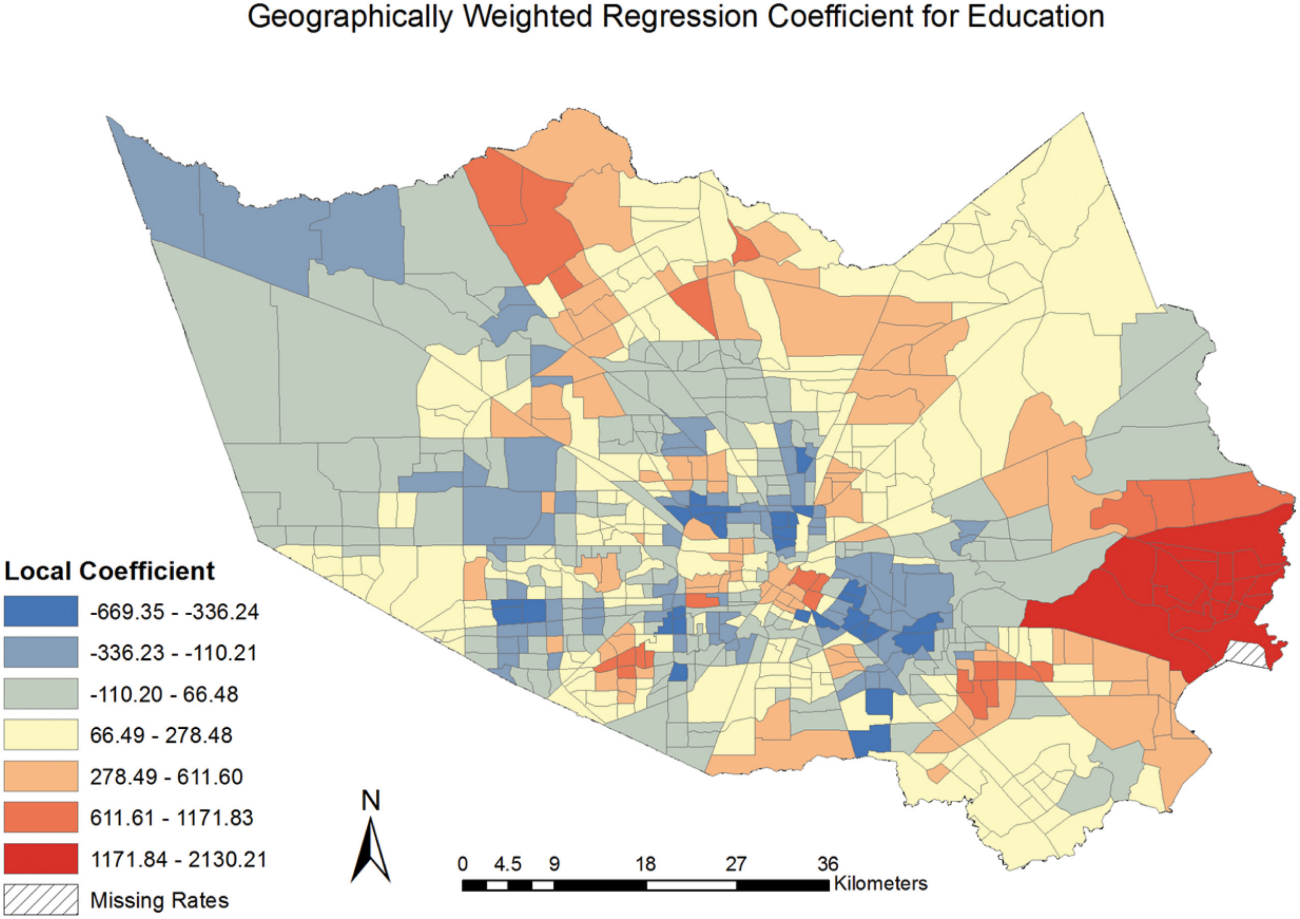
Geographically Weighted Regression Coefficient for Education.

The coefficients for the model covariates were mapped [Figs 6–9] to examine the spatial patterns and variability in their relationship with CVD mortality. All covariates indicated a spatially varying association with CVD mortality. Similar to the pattern seen with social deprivation, though subtly different, the coefficients for the covariates of ages 65 and over and MHHI for black race indicated clustering of high values around the central area of Harris County. [Figs 6 and 7] The local coefficients for MHHI for Asian race and education did not indicate as notable a degree of clustering of the high values, with the variation in the associations more dispersed across Harris County, TX. [Figs 8 and 9]

**Fig 10:**
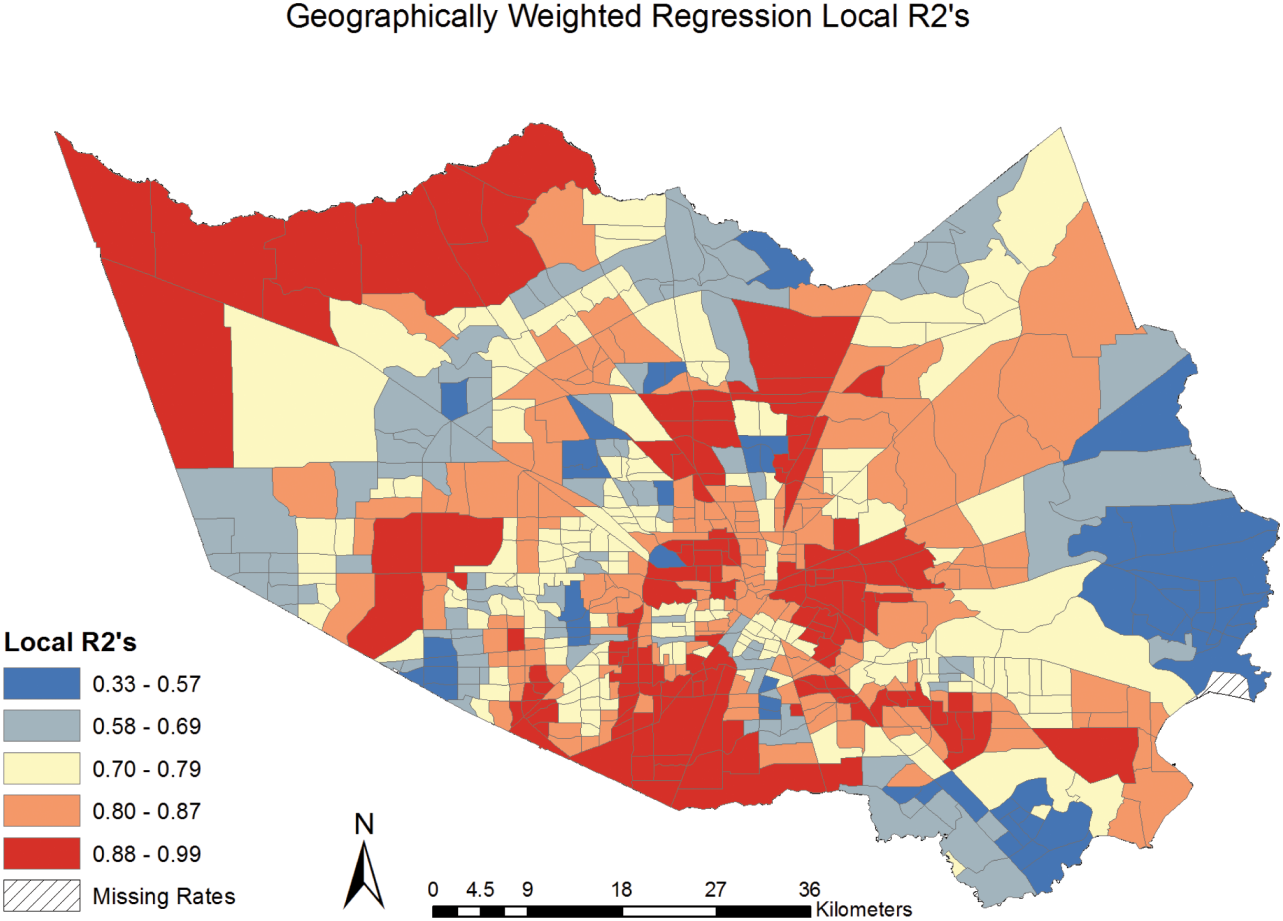
Geographically Weighted Regression Local R2’s.

The Local R^2^ values were mapped, [Fig 10] exhibiting the neighborhoods in Harris County where the covariates explained the greatest proportion of the variance seen in the CVD mortality rates. The R^2^ values range from .33 to .99, indicating the model has a much larger proportion of explained variance in some neighborhoods than in others. Explained variance was relatively high in much of the central and South portions of Harris County, TX. The neighborhoods with lower R^2^ values indicate places where the model would benefit from the inclusion of additional explanatory variables.

The GWR model produced a higher R^2^ than the OLS model (.65 vs. .57) indicating an increase in explained variance by using the GWR model. We can now account for 65% of the variance within the CVD mortality rates with the variables included in the GWR model. The AIC score for the GWR model also decreased, by 57%, from the OLS model (7382.9 for GWR vs. 16736.76 for OLS) further indicating a better model fit with GWR. The residuals from the GWR model were tested for spatial autocorrelation using the Moran’s I statistic, which produced a z-score of -0.51 and a p-value of 0.61, suggesting the spatial pattern does not appear to be significantly different from random and that GWR accounted for spatial autocorrelation.

## Discussion

The present study found a significant (p < .01) and spatially varying association between social deprivation and CVD mortality at the neighborhood-level in Harris County, TX. The results from the GWR model indicated an increase of 25.48 CVD deaths can be expected for each 1-unit increase in the Townsend Deprivation Index, when holding age, median household income by race, and education constant. While the OLS model results demonstrated the statistical association between social deprivation and CVD mortality, the results masked important spatial variations in the relationship. The global Moran’s I test revealed positive and significant (*I* = .11, p < .01) clustering of CVD mortality rates in Harris County, TX, signifying geography is important to account for and supporting the use of the GWR model. The variables included in GWR model accounted for 65% of the variance in CVD mortality rates (versus 57% in the OLS model) and the GWR method successfully accounted for the spatial autocorrelation (z = .51, p = .61) when the studentised residuals were tested with the Moran’s I. These findings revealed notable spatial heterogeneity in the relationship between CVD mortality and social deprivation, [Fig 5] and supports the use of such geographically-informed techniques in future research.

The census-tracts or neighborhoods that experience high levels of social deprivation, as measured by the Townsend Index, simultaneously experience high rates of CVD mortality. This finding is consistent with previous research suggesting that conditions like CVD are exacerbated by neighborhood-level living conditions, which may become internalized biologically and manifest in poorer health outcomes [9,12]. The neighborhoods identified as having the strongest association between CVD mortality and social deprivation may be considered for public health intervention. Despite being a primarily urban area, Harris County (including the City of Houston) has neighborhoods where residents experience social deprivation and likely lack access to important health and social services as a result. The City of Houston, as well as the vast majority of Harris County, is characterized by a lack of zoning and public transportation infrastructure, which contributes to notable urban sprawl and creates a significant reliance on automobile transportation[44,45]. Non-car ownership is one of the four variables comprising the Townsend Index, making this index particularly appropriate for measuring social deprivation in Harris County where non-car-ownership leads to critical mobility issues. Lack of transportation has been well documented as a major barrier to accessing health care and the significant relationship between the Townsend Index CVD mortality in this study further supports this hypothesis [46,47]. This finding underpins importance of community-based health services and the need for resources to be placed directly within neighborhoods that experience access barriers.

The ability to map the local regression coefficients and R^2^‘s produced by the GWR model allow us to better understand geographic differences in the risk for CVD mortality and more accurately inform the placement of needs-based interventions. All variables tested in the model showed different spatial patterns, suggesting that each covariate may be examined separately when tailoring healthcare delivery and programs to a neighborhood. Both social deprivation and other CVD risk factors have significant implications for urbanization, policy, and the built environment. As cities grow, residential segregation by income or race, may be perpetuated by factors associated with social deprivation, and make it more difficult to eliminate health disparities in chronic disease. Long-term exposure to a poor living environment, even as medicine becomes more advanced, may continue to lead to poorer health outcomes in deprived neighborhoods [48,49]. This calls upon the need for urban and city planners to incorporate measures of area deprivation when considering the placement of city services such as housing developments, food retail providers, and healthcare services.

This study builds upon previous spatial research on the effect of geographic health disparities and CVD outcomes. The use of small-area geography, as in this model, remains underutilized by health researchers in the US. Populates are likely more homogenous in terms of socioeconomic status, deprivation, and other relevant neighborhood characteristics in smaller geographic areas [50]. The use of census-tracts rather than a larger area in this study increases the applicability of the findings to community planners or neighborhood-targeted interventions [51]. Additionally, the observed association between CVD mortality and social deprivation emphasizes that health disparities in relation to health and place are broader than just income or race, which are often used as general measures for disparity [52,53] Thus, social deprivation may be a more robust, realistic indicator of community health in regard to the effect of place on CVD outcomes. The spatial variability observed in the association between CVD mortality and the Townsend Index supports previous findings from its use in UK-based studies as well as the future inclusion of the Index in studies looking at social disadvantage, disparities, or health outcomes in the U.S [54–56].

The results from this study may be used to inform the integration of tailored evidence-based prevention and intervention strategies into neighborhoods where the relationship between social deprivation and CVD mortality was largely explained by the model [Figs 3 and 8]. Future studies will want to consider including additional neighborhood characteristics into analyses that may affect the relationship between social deprivation and CVD mortality, such as food deserts, public transportation routes, and accessibility to social and health care services. The application of geographically-informed methods and subsequent results will further the public health field’s ability to accurately target geographic areas and communities where both the outcome and exposure of interest exist.

## Limitations

The study was conducted at a census-tract level, leaving it open to the ecological fallacy by possibly finding association at a population level where little or no association exists on an individual level [57]. Individual behaviors are not accounted for in the model and therefore causal inferences cannot be made regarding the relationship between CVD mortality and social deprivation. To reduce effects of the ecological fallacy, the study used census tracts, which likely made improvements if results were compared to analyses using a larger geographic unit [24]. This study did not account for environmental exposures, such as air pollution, that have been associated with CVD mortality, which could have an effect on the spatial distribution of the disease [35,58]. Similarly, as the study was conducted at a population level, no individual risk factors were associated with CVD mortality, such as alcohol consumption, smoking, or family history. The selection of a deprivation index was limited by available U.S. census-level data, and the availability of more data points could be used to develop a more robust index. GWR models are subject to multicollinearity issues amongst the local estimates in the model, which can limit the allowable covariates in the model. In this study, multicollinearity was assessed prior to using GWR, by assessing VIF in the OLS model, to reduce any issues related to this limitation. It is also possible that GWR may over-fit the model by generating large local coefficients for the GWR model [59]. Finally, the lack of zoning requirements and unique features of Harris County and the City of Houston, TX may produce unique spatial patterns of social deprivation and CVD mortality which may not be generalizable on a larger or different geographic scale. However, the approaches used in this study would readily generalize to other areas of the US, and this study shows the importance of considering geography and social deprivation when considering CVD.

## Conclusions

The strong spatial association between CVD mortality and social deprivation suggests that effects of poverty and social isolation should not be ignored when targeting communities at highest risk for cardiovascular disease morbidity and mortality. Neighborhoods that experience high rates of social deprivation may be also targeted for community-based interventions and services that may reduce the burden of CVD morbidity and mortality. Similarly, social deprivation may need to be recognized and included as a significant CVD risk factor for individuals when being assessed by health care professionals. The findings indicate that significant spatial heterogeneity exists in the relationship between social deprivation and CVD mortality in Harris County, TX, which should be accounted for in future health research. We recommend investigators include spatial and place-based methods to improve understanding of effects of neighborhood-level social deprivation on CVD mortality.

## Supporting Information

S1 Dataset Dataset of Harris County, Census-Tract Variables. (XLSX)

## Abbreviations

AIC: Akaike Information Criterion
CVD: Cardiovascular Disease
GWR: Geographically Weighted Regression
MHHI: Median Household Income
MSA: Metropolitan Statistical Area
OLS: Ordinary Least Squares
TX: Texas
US: United States
VIF: Variance Inflation Factor
